# Identification and validation of diagnostic biomarkers and immune cell abundance characteristics in *Staphylococcus aureus* bloodstream infection by integrative bioinformatics analysis

**DOI:** 10.3389/fimmu.2024.1450782

**Published:** 2024-11-25

**Authors:** Junhong Shi, Li Shen, Yanghua Xiao, Cailing Wan, Bingjie Wang, Peiyao Zhou, Jiao Zhang, Weihua Han, Rongrong Hu, Fangyou Yu, Hongxiu Wang

**Affiliations:** ^1^ Department of Clinical Laboratory, Shanghai Pulmonary Hospital, School of Medicine, Tongji University, Shanghai, China; ^2^ Shanghai Institute of Immunity and Infection, Chinese Academy of Sciences, Shanghai, China

**Keywords:** *Staphylococcus aureus*, bloodstream infection, machine-learning, biomarkers, immune cell abundance

## Abstract

*Staphylococcus aureus* (*S. aureus*) is an opportunistic pathogen that could cause life-threatening bloodstream infections. The objective of this study was to identify potential diagnostic biomarkers of *S. aureus* bloodstream infection. Gene expression dataset GSE33341 was optimized as the discovery dataset, which contained samples from human and mice. GSE65088 dataset was utilized as a validation dataset. First, after overlapping the differentially expressed genes (DEGs) in *S. aureus* infection samples from GSE33341-human and GSE33341-mice samples, we detected 63 overlapping genes. Subsequently, the hub genes including DRAM1, PSTPIP2, and UPP1 were identified via three machine-learning algorithms: random forest, support vector machine-recursive feature elimination, and least absolute shrinkage and selection operator. Additionally, the receiver operating characteristic curve was leveraged to verify the efficacy of the hub genes. DRAM1 (AUC=1), PSTPIP2 (AUC=1), and UPP1 (AUC=1) were investigated and demonstrated significant expression differences (all P < 0.05) and diagnostic efficacy in the training and validation datasets. Furthermore, the relationship between the diagnostic markers and the abundance of immune cells was assessed using cell-type identification by estimating relative subsets of RNA transcripts (CIBERSORT). These three diagnostic indicators also correlated with multiple immune cells to varying degrees. The expression of DRAM1 was significantly positively correlated with B cell naive and mast cell activation, and negatively correlated with NK cells and CD4/CD8^+^ T cells. The expression of PSTPIP2 was significantly positively correlated with macrophage M0, macrophage M1, B cell naive, and dendritic cell activation, while the expression of PSTPIP2 was negatively correlated with NK cells and CD4/CD8^+^ T cells. Significant negative correlations between UPP1 expression and T cell CD4 memory rest and neutrophils were also observed. Finally, we established a mouse model of *S. aureus* bloodstream infection and collected the blood samples for RNA-Seq analysis and RT-qPCR experiments. The analysis results in RNA-Seq and RT-qPCR experiments further confirmed the significant expression differences (all P < 0.05) of these three genes. Overall, three candidate hub genes (DRAM1, PSTPIP2, and UPP1) were identified initially for *S. aureus* bloodstream infection diagnosis. Our study could provide potential diagnostic biomarkers for *S. aureus* bloodstream infection patients.

## Introduction

The opportunistic pathogenic bacterium *Staphylococcus aureus* has successfully adapted to the human body’s environmental conditions (1). It causes a spectrum of infections in communities and hospitals, ranging from skin and soft tissue infections to life-threatening bloodstream infections (2). A critical feature of bloodstream infections by *S. aureus* is the coordinated and timely expression of virulence factors and other relevant genes by the pathogen. Due to its prevalence, *S. aureus* ranks among the leading pathogens causing bloodstream infections (3). The *S. aureus* bloodstream infections are characterized by high mortality rates (ranging from 20% to 50%), frequent recurrence (5-10%), and sustained injury in over one-third of survivors (3–5). Over the past three decades, the incidence rate of *S. aureus* bloodstream infection has been increasing in developed countries (3, 4), but remains a significant but often overlooked issue in developing countries (6). Therefore, there is an urgent need to identify biomarkers for the diagnosis of *S. aureus* bloodstream infection.

During infections, *S. aureus* could trigger inflammatory responses, including the secretion of cytokines and chemokines that recruit leukocytes to the area of infection. These recruited neutrophils, monocytes, macrophages, NK cells, Dendritic cells (DCs), and CD4/CD8^+^ T cells play crucial roles in both the direct killing of bacteria and the indirect control of infection, such as contributing to the cytokine milieu, clearing damaged cells, and presenting antigen to initiate adaptive immunity (7). Hence, discovering new immunological biomarkers was important not only for the diagnosis but also for the application of immunotherapy in *S. aureus* bloodstream infection.

High-throughput sequencing was a valuable method for investigating changes in disease gene expression and distinguishing possible disease-related genes for new diagnostic and therapeutic biomarkers (8). Gene expression levels serve as essential indicators for diagnosing various disorders, including *S. aureus* bloodstream infection (9). Machine learning method assists in assessing high-dimensional transcriptome data and identifying biologically significant genes (10).

In this study, we integrated multiple high-throughput sequencing datasets of *S. aureus* bloodstream infections and employed machine learning algorithms for the first time to identify three characteristic genes associated with these infections, distinguishing our work from previous studies (8, 11–13). In addition, using a mouse model of *S. aureus* bloodstream infection, we validated the diagnostic value of these three genes through RNA-Seq and RT-qPCR experiments. Additionally, we investigated the relationship between diagnostic markers and immune cell abundance to acquire a more in-depth understanding of the molecular immune mechanisms underlying *S. aureus* bloodstream infections. Our study may offer potential diagnostic biomarkers and select potential candidates receiving immunotherapy for patients with *S. aureus* bloodstream infection.

## Methods

### Public gene expression datasets

Accessing the Gene Expression Omnibus (GEO) database (https://www.ncbi.nlm.nih.gov/geo/), which is a public collection of high-throughput gene expression data, chips, and microarrays, was how the information was collected. We searched the GEO database with the keywords “*Staphylococcus aureus*” [MeSH Terms] AND “Bloodstream infection”[All Fields]. None of the included samples were associated with any other diseases. The sample size of both the pediatric sepsis group and the normal group was greater than 10. Finally, GSE33341 (14) was utilized as the discovery dataset, which contained samples from human and mice. Another dataset GSE65088 (15) was applied as a validation dataset.

### Identification of the differentially expressed genes

The Wilcoxon test was utilized to identify differentially expressed genes (DEGs) between the *S. aureus* bloodstream infection group and the control group. A volcano plot was generated to visualize the differential expression of DEGs. A P value < 0.05 and |log2FC|> 1 were considered to be the cutoffs for DEGs.

### Evaluation of functional enrichment

Gene Ontology (GO) and the Kyoto Encyclopedia of Genes and Genomes (KEGG) pathway enrichment analyses were conducted via the “clusterProfiler” (16) package in R to explore possible biological features of DEGs. Gene set enrichment analysis (GSEA) (17) was also used to investigate the enrichment pathways via the “clusterProfiler” package in R.

### Screening and validation of diagnostic markers

Firstly, we intersected the up-regulated genes in the human *S. aureus* infection group and the mice *S. aureus* infection group in GSE33341 to obtain genes associated with *S. aureus* bloodstream infection. Subsequently, these genes were further screened using three machine-learning algorithms, random forests (RF), support vector machine-recursive feature elimination (SVM-RFE), and least absolute shrinkage and selection operator (LASSO) logistic regression, to identify robust biomarkers for *S. aureus* bloodstream infection. The “randomForest” R package in R was used to implement the random forest technique with 100 trees generated for each datapoint, and genes with top MeanDecreaseAccuracy were screened out (18). LASSO logistic regression investigation was conducted with the R package “glmnet”, and minimal lambda was considered optimal. In our study, the selection of optimization parameters was cross-verified by a factor of 10, and the partial likelihood deviation met the minimum criteria (19). The DEGs were also determined by applying a support vector machine recursive feature elimination (SVM-RFE) algorithm based on a nonlinear SVM using R package “kernlab”, “e1071”, and “caret” (20). It was evaluated based on the study of receiver operating characteristic (ROC) curves, and the area under the curve (AUC) was calculated to assess the predictive capability of these markers. The GSE65088 dataset was enrolled to validate the predictive power of these biomarkers.

### Assessment of immune cell abundance

Immune cell abundance was assessed by computing the differential abundances of 22 immune cells using the CIBERSORT (21) algorithm. The correlation between gene expression and immune cells were assessed using Pearson’s correlation coefficients. Correlation plots were plotted using the “ggpubr” R package.

### Construction of *S. aureus* bloodstream infection

Methicillin-sensitive *Staphylococcus aureus* (MSSA) Newman strain was grown for 16 h on TSB medium at 37°C. Overnight cultures were centrifuged at 2683g (RCF) for 5 min at room temperature and adjusted to a concentration of 2 × 10^9^ CFU/mL using phosphate-buffered saline (PBS). Next, injected into female Balb/c mice via the tail vein with 100 μL PBS containing 2 × 10^8^ CFU bacterial cells suspended. At 8 h.p.i., anesthetized the mice with 2,2,2-tribromoethanol (5 mg/25 g). Used surgical scissors to remove mouse whiskers, then clamped the eyeball with tweezers and quickly removed it, allowing blood to flow from the eye socket into the EP tube. The blood sample was immediately placed in liquid nitrogen and maintained at −80°C until RNA extraction.

### RNA-Seq and data processing

After the blood samples were collected from mice infected with *S. aureus*, the RNA for RNA-Seq samples were immediately mixed with Trizol Reagent (Ambion^®^) and then sent to Shanghai Personal Biotechnology Cp. Ltd for the subsequent RNA transcriptome sequencing work. Following library preparation and pooling of different samples, the samples were subjected to Illumina sequencing. Commonly, the RNA-Seq use PE150 (paired-end 150nt) sequencing. Raw data (raw reads) of FASTQ format were first processed through in-house perl scripts. In this step, clean data (clean reads) were obtained by removing the following reads: (1) reads with adapter; (2) reads with more than 3 N; (3) reads with more than 20% nucleotides with Qphred<=5; At the same time, Q20, Q30 and GC content of the clean data were calculated. Then, map the clean reads to the silva database to remove the rRNA. All the downstream analyses were based on clean data without rRNA. Paired-end clean reads were aligned to the reference genome using Hisat2 (22). Featurecount (23) was used to count the reads numbers mapped to each gene.

### RNA extraction and real-time polymerase chain reaction

The total RNA of blood was isolated by Trizol Reagent (24) and then was reverse transcribed into cDNA using the PrimeScript RT reagent kit with gDNA Eraser (Takara). Real-time quantitative PCR (RT-qPCR) was performed using TB GreenTM Premix Ex TaqTM II (Takara) on QuantStudioTM 5 Real-Time PCR System (Applied Biosystems). RNA expression levels of DRAM1, PSTPIP2, and UPP1 genes unified to GAPDH were calculated by the formula 2^−ΔΔCt^. All primers used in this study were listed in **Supplementary Table 1**. Each reaction was performed trice.

### Statistical analysis

R version 4.2.2 was utilized for all statistical analyses and graphics except for RT-qPCR results which were analyzed by GraphPad Prism 8 (GraphPad Software Inc. San Diego, CA, USA). Statistical significance was determined by a two-tailed test with a P value of less than 0.05. **P < 0.01, ****P < 0.0001.

## Results

### Screening of DEGs in *S. aureus* bloodstream infection

**Figure 1:**
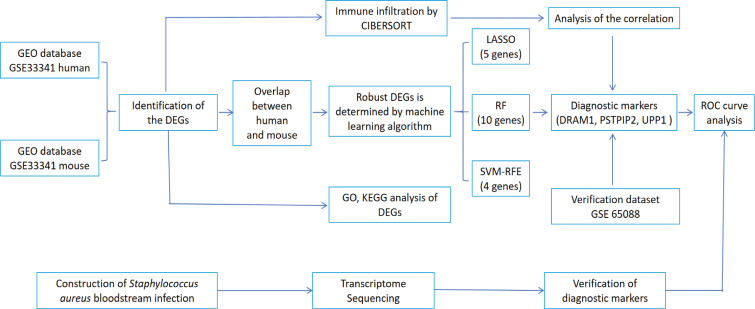
The flowchart depicting the investigation procedure. GEO, gene expression omnibus; GSEA, gene set enrichment analysis; CIBERSORT, cell-type identification by estimating relative subsets of RNA transcripts; DEGs, differentially expressed genes; GO, gene ontology; KEGG, Kyoto Encyclopedia of Genes and Genomes; LASSO, Least absolute shrinkage and selection operator; RF, random forest; SVM-RFE, support vector machine-recursive feature elimination; ROC, receiver operating characteristic curve.

**Figure 2:**
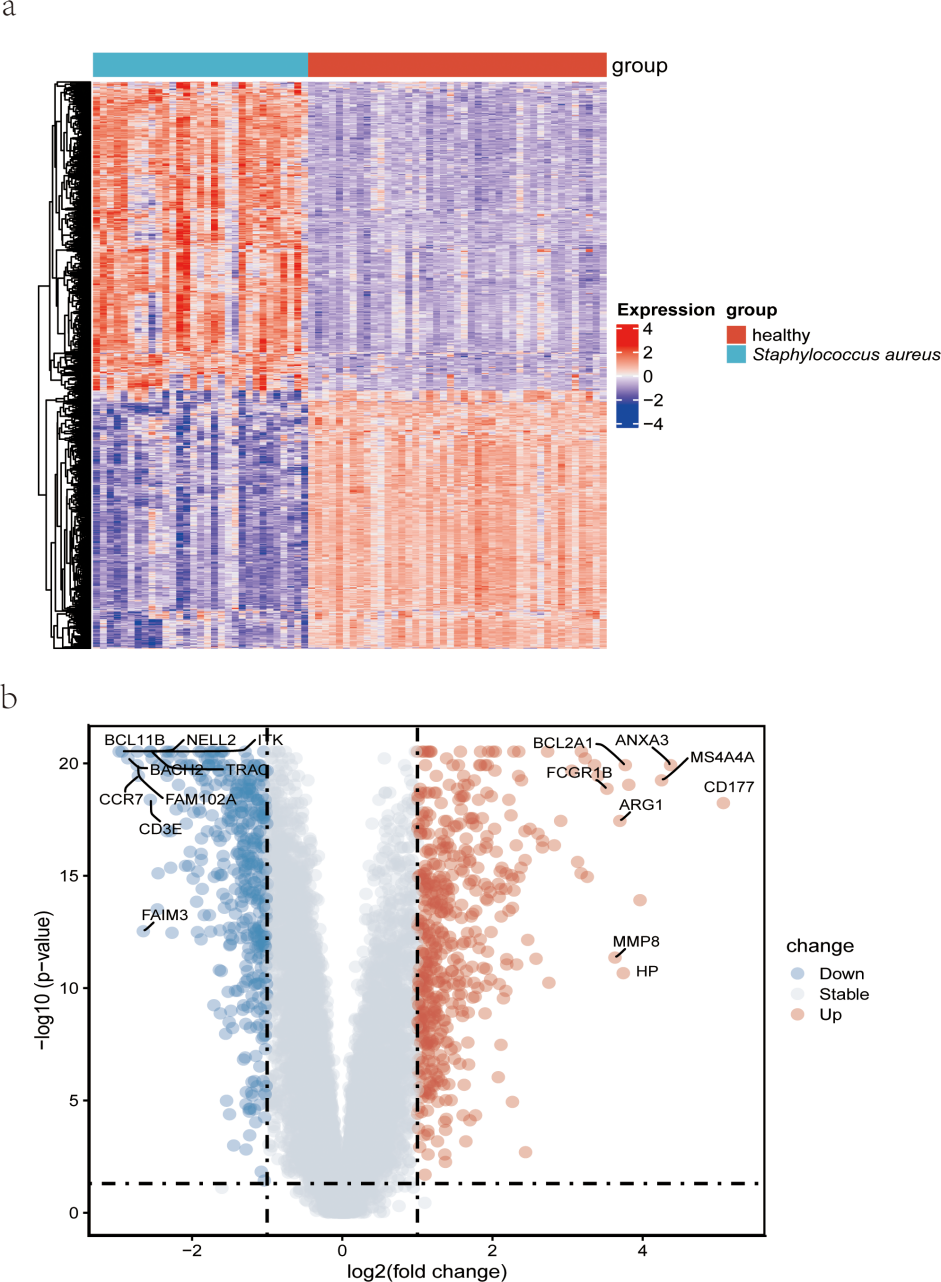
Detection of differentially expressed genes from datasets GSE33341 on *S. aureus* patients. **(A)** A heatmap comparing the genes that were differentially expressed in *S. aureus* bloodstream patients and control patients; **(B)** Volcano plot of the 482 DEGs. DEGs, differentially expressed genes; FC, fold-change.

The clinical characteristics of the two groups of samples are presented in **Supplementary Table 2**. **Figure 1** displays the study design of this research. The human blood samples in GSE33341, consisting of 31 *S. aureus* infection samples and 43 control samples, were applied to obtain 482 DEGs. Following the identification of DEGs, heatmap (**Figure 2A**) and volcano plots (**Figure 2B**) were drawn to present these findings.

### Functional enrichment analysis of DEGs

**Figure 3:**
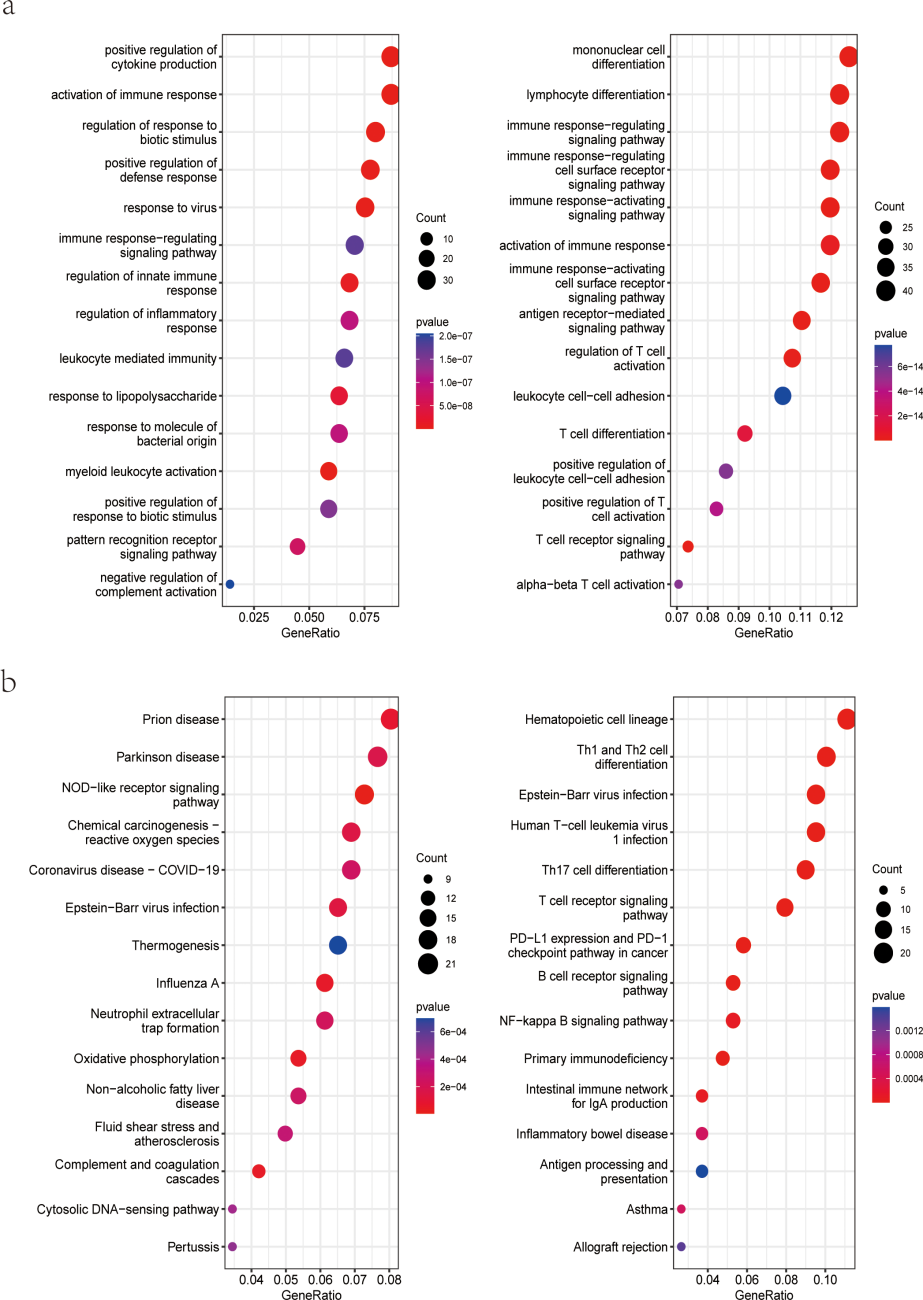
Functional enrichment analysis of DEGs. **(A)** GO enrichment analysis of upregulated (left) and downregulated (right) genes in the dataset GSE33341 human group; **(B)** KEGG pathway enrichment analysis of upregulated (left) and downregulated (right) genes in the dataset GSE33341 human group. GO, Gene Ontology; KEGG, Kyoto Encyclopedia of Genes and Genomes.

Functional analysis was performed to gain a more thorough understanding of the biological functions of these DEGs. GO enrichment analysis showed that up-regulated DEGs were related to positive regulation of cytokine production and activation of the immune response (**Figure 3A**, left). Down-regulated DEGs were enriched in mononuclear cell differentiation, lymphocyte differentiation, and immune response-regulating signaling pathway (**Figure 3A**, right). Likewise, KEGG analysis for up-regulated DEGs was associated with Prion disease, Parkinson disease, and NOD-like receptor signaling pathway (**Figure 3B**, left). KEGG analysis for down-regulated DEGs was enriched in hematopoietic cell lineage, Th1 and Th2 cell differentiation, and Th17 cell differentiation (**Figure 3B**, right).

### Screening and validation of diagnostic markers

**Figure 4:**
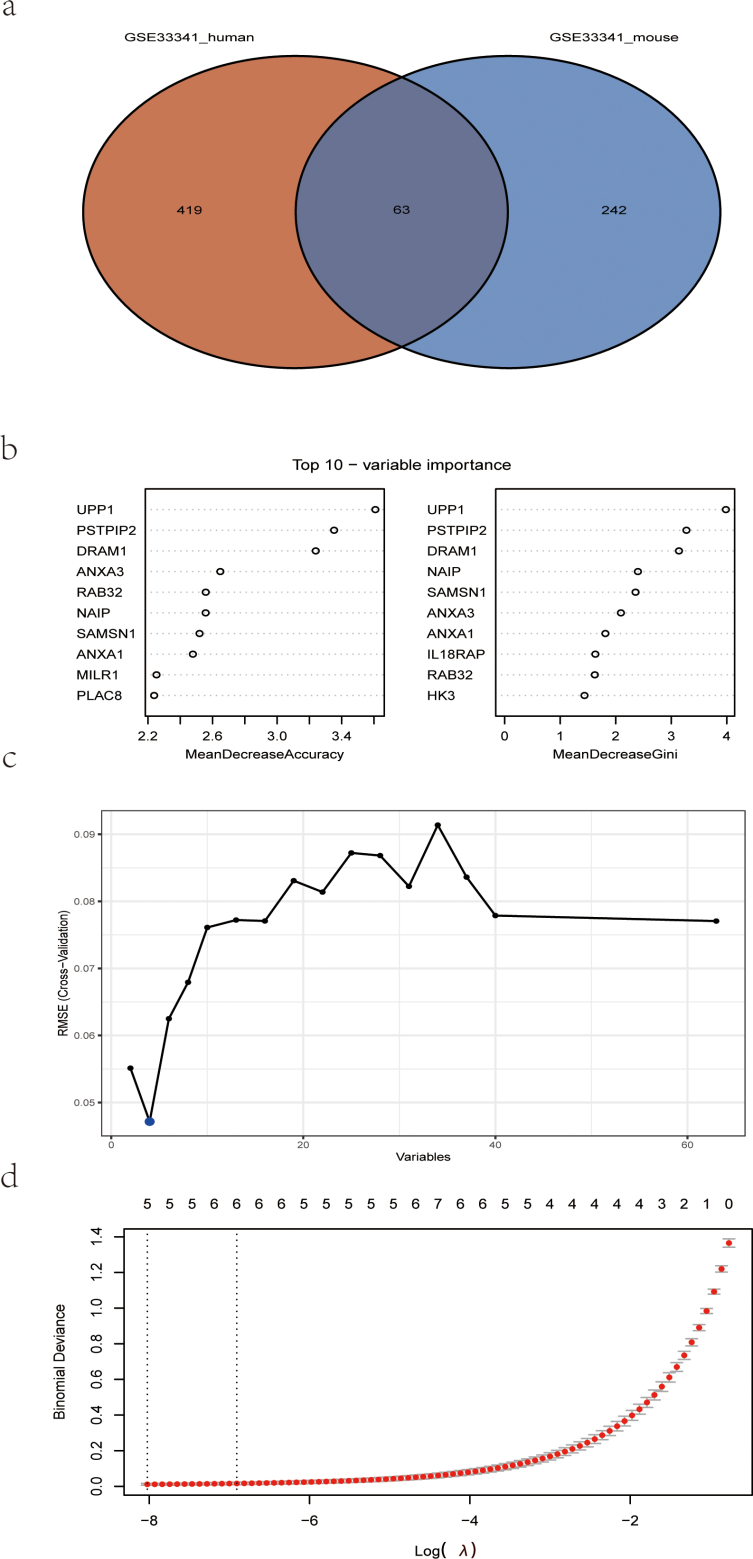
Detection of diagnostic markers using a thorough method. **(A)** Venn diagram of upregulated genes of human *S. aureus* infection versus mice group in GSE33341; **(B)** based on RF algorithm to screen biomarkers; **(C)** Based on SVM-RFE to screen biomarkers; **(D)** LASSO logistic regression algorithm to screen diagnostic markers. RF, random forest; SVM-RFE, support vector machine-recursive feature elimination; LASSO, least absolute shrinkage, and selection operator.

**Figure 5:**
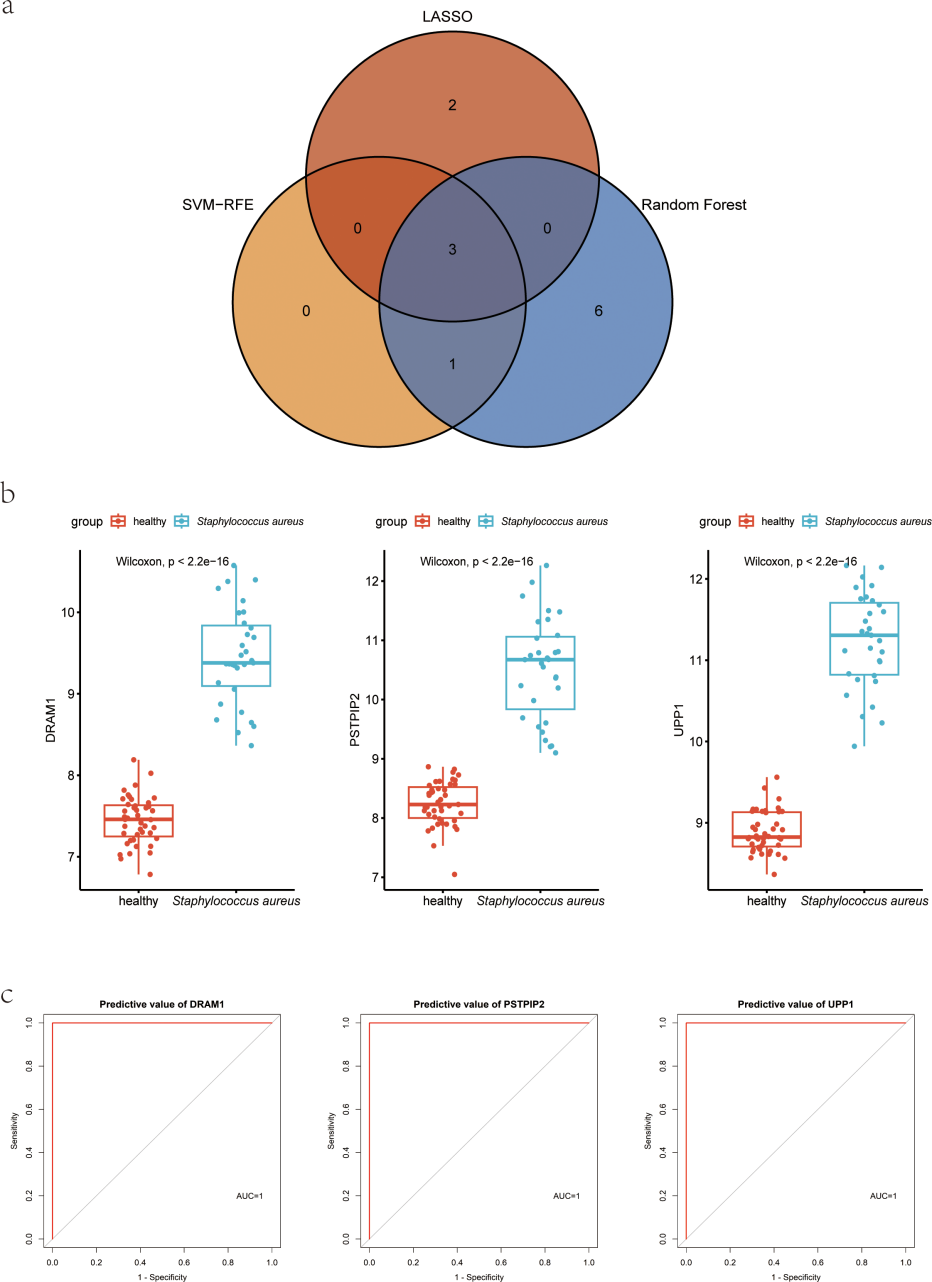
Hub genes for *S. aureus* blood infection diagnosis. **(A)** Venn diagram showed the intersection of diagnostic markers obtained by the three algorithms; **(B)** Boxplot showed the expression of hub genes between the *S. aureus* infection group and control group in discovery dataset GSE33341 human group; **(C)** The ROC curve of the diagnostic efficacy verification between the *S. aureus* infection group and control group in discovery dataset GSE33341 human group.

The human blood samples in GSE33341, consisting of 31 *S. aureus* infection samples and 43 control samples, and the mice blood samples in GSE33341, including 10 *S. aureus* infection samples and 21 control samples, were exploited for analyzing the up-regulated genes separately. There were 482 up-regulated DEGs in the GSE33341-human samples and 305 up-regulated DEGs in the GSE33341-mice samples. A Venn plot was drawn to present the up-regulated genes intersected by GSE33341-human and GSE33341-mice samples (**Figure 4A**). Then we adopted three machine-learning algorithms to identify feature genes: Random Forest selected the top 10 genes (**Figure 4B**); SVM-RFE screened 4 genes (**Figure 4C**) and LASSO regression analysis was utilized to select 3 predicted genes from among the statistically significant univariate variables (**Figure 4D**). The three algorithms finally identified DRAM1, PSTPIP2, and UPP1 as the diagnostic markers (**Figure 5A**).

In the GSE33341, these three genes not only were highly expressed in the *S. aureus* infection group but also presented with good discriminative power between *S. aureus* and the control group (**Figure 5B**; **Supplementary Figure 1**). ROC curves for DRAM1, PSTPIP2, and UPP1 also highlighted them as potential diagnostic biomarkers (**Figure 5C**).

Meanwhile, we also acquired another independent cohort with *S. aureus* infection to validate the above findings. In GSE65088, the significantly high expression for DRAM1, PSTPIP2, and UPP1 in *S. aureus* group was also observed (**Supplementary Figure 2A**), along with high AUC values (**Supplementary Figure 2B**), which indicates that the biological markers had high predictive value accuracy.

### Association of biomarkers with immune cells abundance

**Figure 6:**
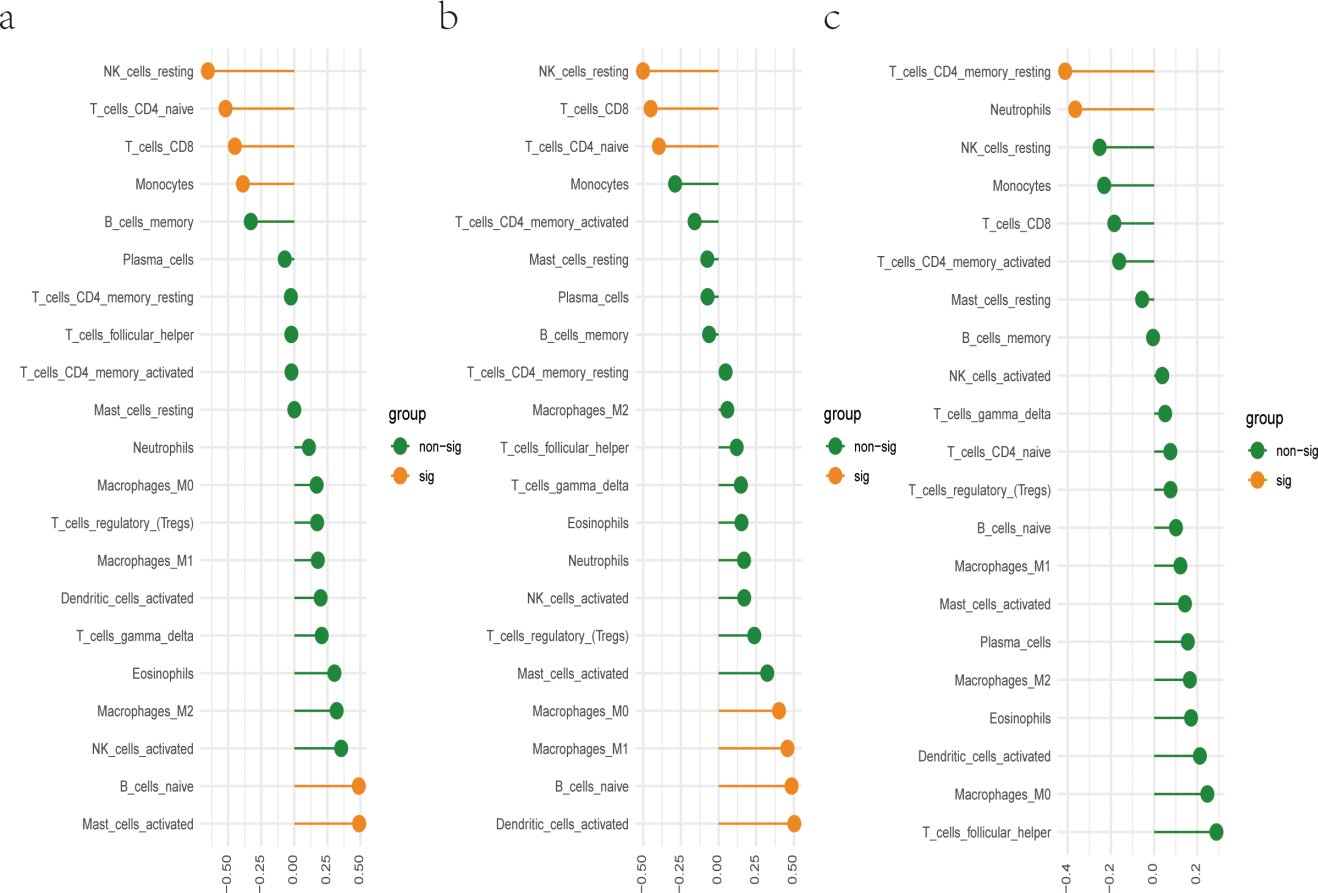
There is a correlation between hub genes and immune cells. **(A)** Correlation between DRAM1 and immune cells; **(B)** Correlation between PSTPIP2 and immune cells; **(C)** Correlation between UPP1 and immune cells.

CIBERSORT algorithm was utilized to evaluate the immune cell abundance. Based on a correlation analysis, we assessed the relationship between immune cells and three diagnostic biomarkers. We found DRAM1 was significantly positively associated with B cell naive and Mast cell activated. However, the expression of DRAM1 was negatively associated with NK cells and CD4/CD8^+^ T cells (**Figure 6A**). Meanwhile, PSTPIP2 was notably positively correlated with Macrophages M0, Macrophages M1, B cell naive, and Dendritic cells activated, the expression of PSTPIP2 was negatively associated with NK cells and CD4/CD8^+^ T cells (**Figure 6B**). Furthermore, UPP1 was remarkably negatively related to T cells CD4 memory resting and Neutrophils (**Figure 6C**). The above results of analysis suggested there was a potential connection between these three biomarkers and a wide variety of immune cells. Immunological prophylaxis and therapy for *S. aureus* are attractive goals. Our findings provided reasonable application of these markers for screening potential patients with *S. aureus* bloodstream infection for immunotherapy.

### Validation of diagnostic markers using RNA-Seq and RT-qPCR for *S. aureus* bloodstream infection mice models

**Figure 7:**
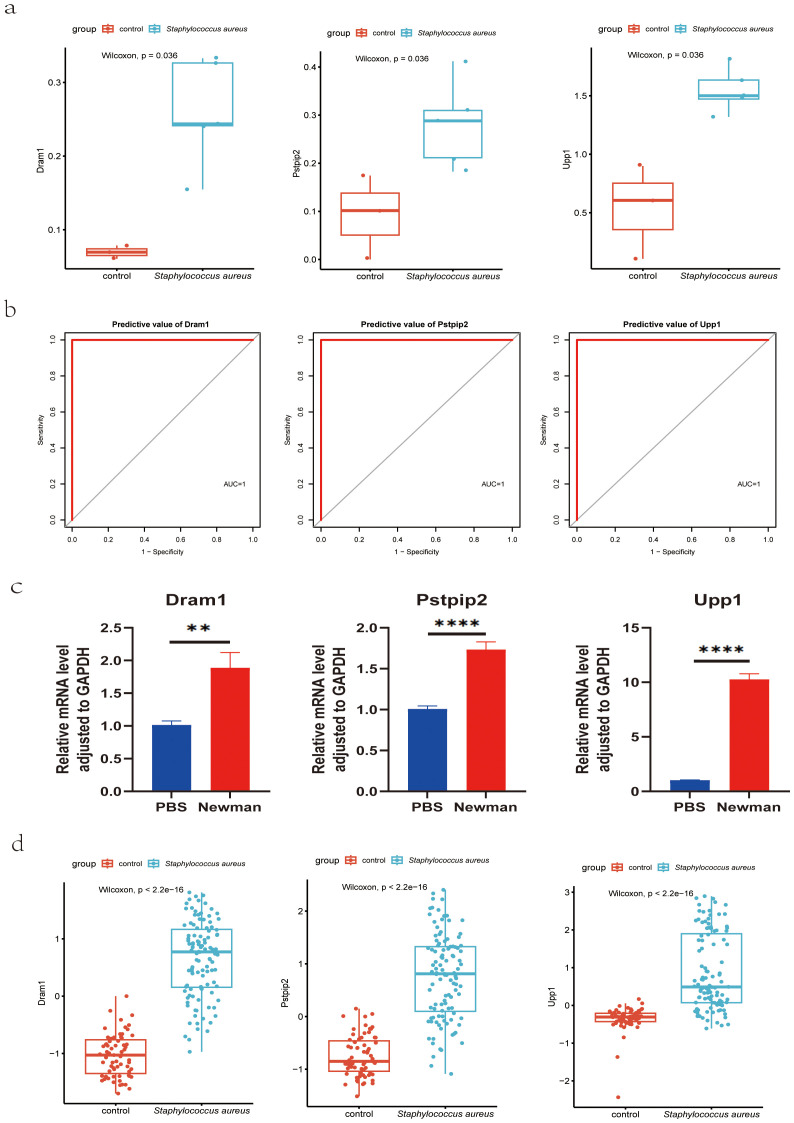
Validation of hub genes for *S. aureus* blood infection diagnosis. **(A)** Validation of diagnostic markers using RNA-Seq; **(B)** The ROC curve of the diagnostic efficacy verification in RNA-Seq analysis; **(C)** Validation of diagnostic markers using RT-qPCR; **P < 0.01 and ****P < 0.0001. d Validation of diagnostic markers via the combined mouse sequencing data including GSE33341 mice and blood infection mouse model sequencing data. AUC, area under the curve; ROC, receiver operating characteristic curve.

To further confirm the diagnostic value of these three genes, we constructed a mice model of *S. aureus* bloodstream infection and collected the blood of mice for RNA-Seq analysis and RT-qPCR experiments. The reason we chose the *S. aureus* Newman strain was that it is a hypervirulent stain that has been widely applied in the various models of *S. aureus*. We isolated the total RNA of the blood and synthesized the cDNA. RNA-Seq analysis results confirmed the above results (**Figure 7A**), with ROC results showing the high diagnostic value for these three genes (**Figure 7B**). RT-qPCR results further validated that the RNA expression levels of DRAM1, PSTPIP2, and UPP1 genes were significantly increased in the *S. aureus* Newman tread group compared to the control group (**Figure 7C**). Meanwhile, these three genes were significantly higher in *S. aureus* group in the combined mouse dataset including GSE33341 mice and blood infection mouse model sequencing data (**Figure 7D**).

## Discussion

The opportunistic pathogen *S. aureus* adapted to human hosts, could result in fatal bloodstream infection (25). It represented a heterogeneous clinical entity with a high risk of metastatic complications and a high in-hospital mortality rate of 20% to 30%. Optimized diagnostic and therapeutic approaches can improve patients’ outcomes (26). The Agr quorum-sensing system, one of the earliest regulators discovered to be involved in *S. aureus* bloodstream infections, is essential for the secretion of numerous toxins and other soluble virulence factors (27). Another virulence regulatory system closely related to *S. aureus* bloodstream infections is the two-component ArlRS system, and its downstream effector, the global regulator MgrA (27). Under the background of *S. aureus* bloodstream infections, this cascade reaction was shown to regulate plasma aggregation, adhesion, and interactions with endothelium (28, 29). Furthermore, the ArlRS-MgrA cascade regulates the expression of several immune evasion genes to evade host defense (30–32). These findings underscore *S. aureus’s* ability to cause bloodstream infection by expressing a series of virulence genes, emphasizing the urgency of finding biomarkers for bloodstream infection of *S. aureus* for early diagnosis and treatment.

In recent years, extensive studies have attempted to discover diagnostic biomarkers for *S. aureus* bloodstream infections. Erin et al. found that *S. aureus* induced a muted host response in human blood that blunts the recruitment of neutrophils to promote the survival of pathogens during invasive infection (33). Sun et al. constructed a predictive model for sepsis in children with *S. aureus* bloodstream infections, which could guide clinicians in optimizing the treatment plan according to these risk factors and drug sensitivity results for minimizing unnecessary invasive procedures (34). Rachel et al. found that manipulation of autophagy in phagocytes facilitated *S. aureus* bloodstream infection (35). Sinead et al. carried out a prospective study in 61 patients with *S. aureus* bloodstream infection and revealed that IL-6 might be an early inflammatory marker of complicated *S. aureus* bloodstream infection (36). However, these diagnostic biomarkers more or less suffer from some limitations. Identifying new diagnostic markers for *S. aureus* bloodstream infection was urgently needed.

In this study, we attempted to identify new diagnostic biomarkers for *S. aureus* bloodstream infection. First, we identified the up-regulated genes in the *S. aureus* infection group common to the GSE33341-human and GSE33341-mouse datasets. Subsequently, the hub genes including DRAM1, UPP1, and PSTPIP2 were certificated by the use of three machine-learning algorithms. Further, we verified the findings by another dataset GSE65088, and developed a mice model of *S. aureus* bloodstream infection to collect the blood of mice for RNA-Seq analysis and RT-qPCR experiments. The receiver operating characteristic curve was employed to verify the efficacy of the hub genes. To summarize, our results suggest that DRAM1, UPP1, and PSTPIP2 were potential *S. aureus* bloodstream infection diagnostic indicators.

Currently, many studies have focused on the three genes mentioned above in *S. aureus* or other bacterial infections. DNA damage-regulated autophagy modulator 1 (DRAM1) is a stress-inducible regulator of autophagy and cell death (37). Xie et al. confirmed that DRAM1 could independently promote the zebrafish host defense against *Mycobacterium marinum (* 38), and its role in facilitating Lysosomal Delivery of *Mycobacterium marinum* in murine RAW264.7 macrophages (39), suggesting DRAM1 is a host resistance factor against intracellular mycobacterial infection. Similarly, Zhang et al. demonstrated that deficiency in DRAM1 exacerbated pyroptotic cell death of Mycobacteria-infected macrophages (40). Han et al. found that DRAM1 expression was up-regulated in the *S. aureus*-treated bovine mammary epithelial cells and triggered the production of autophagosome (41). This appeared to coincide with our results since we demonstrated that DRAM1 was highly expressed in the blood of mice infected with *S. aureus*. Sun et al. proved upregulation of DRAM1 was involved in regulating autophagy and glycolysis in C10_ULK1 cells in response to both *Escherichia coli* (*E. coli*) infection and E. coli sepsis (42). Uridine phosphorylase 1 (UPP1) encodes uridine phosphorylase, a key enzyme that participates in the regulation of intracellular uridine homeostasis and the metabolism of pyrimidine ribonucleosides (43). Fan et al. revealed that UPP1 emerged with remarkable diagnostic value in pediatric septic shock and was involved in immune cell infiltration (44). Similarly, Lai et al. analyzed GEO datasets and found that UPP1 was upregulated in the sepsis group, and confirmed this finding by establishing a sepsis-induced acute lung injury model (45). Our research further expanded the diagnostic value of UPP1 and indicated its likelihood as a diagnostic biomarker. Proline-serine-threonine phosphatase Interacting Protein 2 (PSTPIP2), also known as macrophage F-actin–associated and tyrosine-phosphorylated protein (MAYP), is a Fes CIP4 homology domain (FCH) and Bin/Amphiphysin/Rvs (BAR; F-BAR) protein, predominantly expressed in the myeloid lineage (46). Johnny et al. uncovered that PSTPIP2 was highly expressed in the confirmed bacterial infection patients, correlating with infection status (47). Chen et al. validated the high expression of PSTPIP2 in patients infected with *E. coli* by analyzing GEO datasets and ex-vivo human blood models (48).

Additionally, the relationship between these diagnostic markers and infiltrating immune cells was further studied. The changes in various immune cell infiltration may be relevant to the occurrence and progression of *S. aureus* bloodstream infection (33, 49). NK cells are pivotal in the first line of defense of the human immune system (50, 51). They mediated some immune responses during anti-tumor and various viral infections and were the “natural barrier” in the human immune system (52). Under the stimulation of LPS and so on, dormant macrophages (M0) could induce polarization into M1 type macrophages, secreting a large number of pro-inflammatory factors, including IL-1, IL-6, and TNF-α, to promote inflammation, bacterial killing, and phagocytosis (53). M2 macrophages were mainly activated by IL-4 inflammatory factors and inhibit M1 macrophages by secreting anti-inflammatory cytokines such as IL10 (54). Neutrophils are one of the important cells in the immune system, with various functions, including chemotactic, regulatory, phagocytic, degranulation, and bactericidal effects (55, 56). Dendritic cells could uptake, process, and present antigens, and were initiators of adaptive immune responses (57, 58). CD4^+^ T cells mainly recognized foreign antigens presented by antigen-presenting cells (APCs) and generated responses (59). CD8^+^ T cells cloud secrete cytokines including TNF-α, IFN-γ, and the production and release of cytotoxic particles to defend against intracellular viruses and bacteria (60). In our research, DRAM1 expression was significantly positively correlated with B cell naive and mast cell activation, and negatively correlated with NK cells and CD4^+^/CD8^+^ T cells. PSTPIP2 expression was significantly positively correlated with macrophage M0, macrophage M1, B cells naive, and dendritic cells, while negatively correlated with NK cells and CD4/CD8^+^ T cells. UPP1 expression showed significant negative correlations with T cell CD4 memory rest and neutrophils. Li et al. (61) associated PD-1/PD-L1 signaling with the immunosuppressive state in *S. aureus* osteomyelitis, suggesting potential novel therapies combining PD-1/PD-L1 blockade with antibiotics for the treatment of *S. aureus* osteomyelitis. Therefore, our proposed diagnostic biomarkers may also be used to select potential patients with *S. aureus* bloodstream infection for the utilization of immunotherapy.

However, this study also had some limitations. Despite having reported the diagnostic value of these three markers in *S. aureus* bloodstream infections, unfortunately, we have not yet collected blood samples from patients with *S. aureus* bloodstream infection, which is a limitation of our study. Additionally, the *S. aureus* bloodstream infection-related molecular mechanisms should be investigated further by constructing animal models and cellular experiments. Meanwhile, the differences in immune response between mice and humans might affect the translational relevance of our findings.

Here, our study initially identified that DRAM1, PSTPIP2, and UPP1 were potential diagnostic indicators for *S. aureus* bloodstream infection. Furthermore, these three diagnostic genes also correlate with multiple immune cells to varying degrees and may be used for the *S. aureus* selection of potential patients for the utilization of immunotherapy.

## Data Availability

The raw data has been uploaded to the NCBI database (accession number: PRJNA1171428).

## Supplementary material

Supplementary Table 1 Primer pairs used in RT-PCRs.

Supplementary Table 2 Information on microarray datasets obtained from GEO.
