# Supplementary material for: Identification and validation of diagnostic biomarkers and immune cell abundance characteristics in Staphylococcus aureus bloodstream infection by integrative bioinformatics analysis
Source: Front Immunol. 2024 Nov 25;15:1450782. doi: 10.3389/fimmu.2024.1450782 (PMC11626409; doi:10.3389/fimmu.2024.1450782)
Supplement: Supplementary file 3 [file Presentation1.pdf]

Supplementary Figure 1

Hub genes for *S. aureus* blood infection diagnosis. a Boxplot showed the expression of hub genes between *S. aureus* infection group and control group in the discovery dataset GSE33341 mice group; b The ROC curve of the diagnostic efficacy verification between *S. aureus* infection group and control group in the discovery dataset GSE33341 mice group

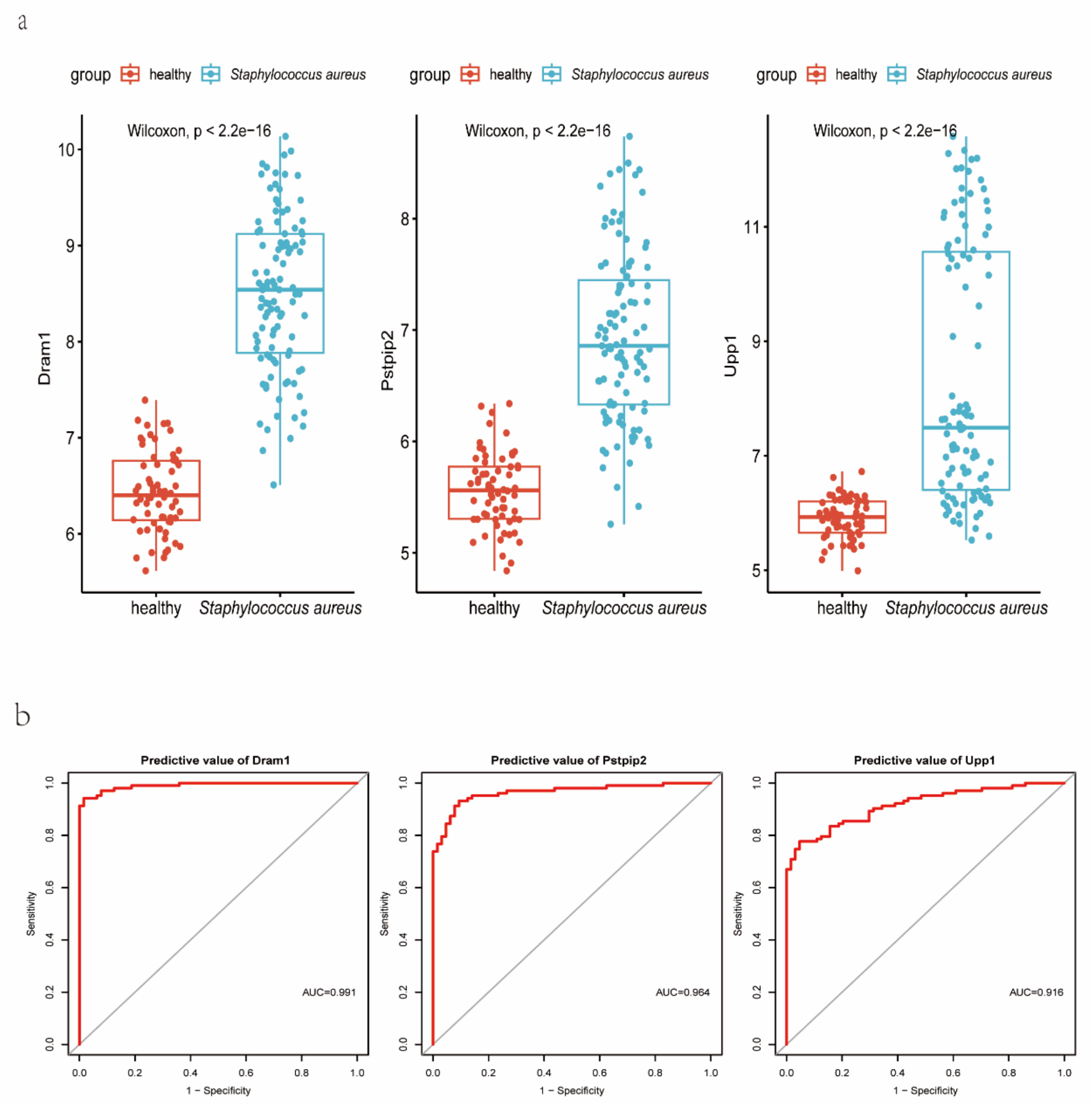

Supplementary Figure 2

Hub genes for *S. aureus* blood infection diagnosis. a Boxplot showed the expression of hub genes between the *S. aureus* infection group and control group in validation dataset GSE65088; b The ROC curve of the diagnostic efficacy verification between the *S. aureus* infection group and control group in validation dataset GSE65088

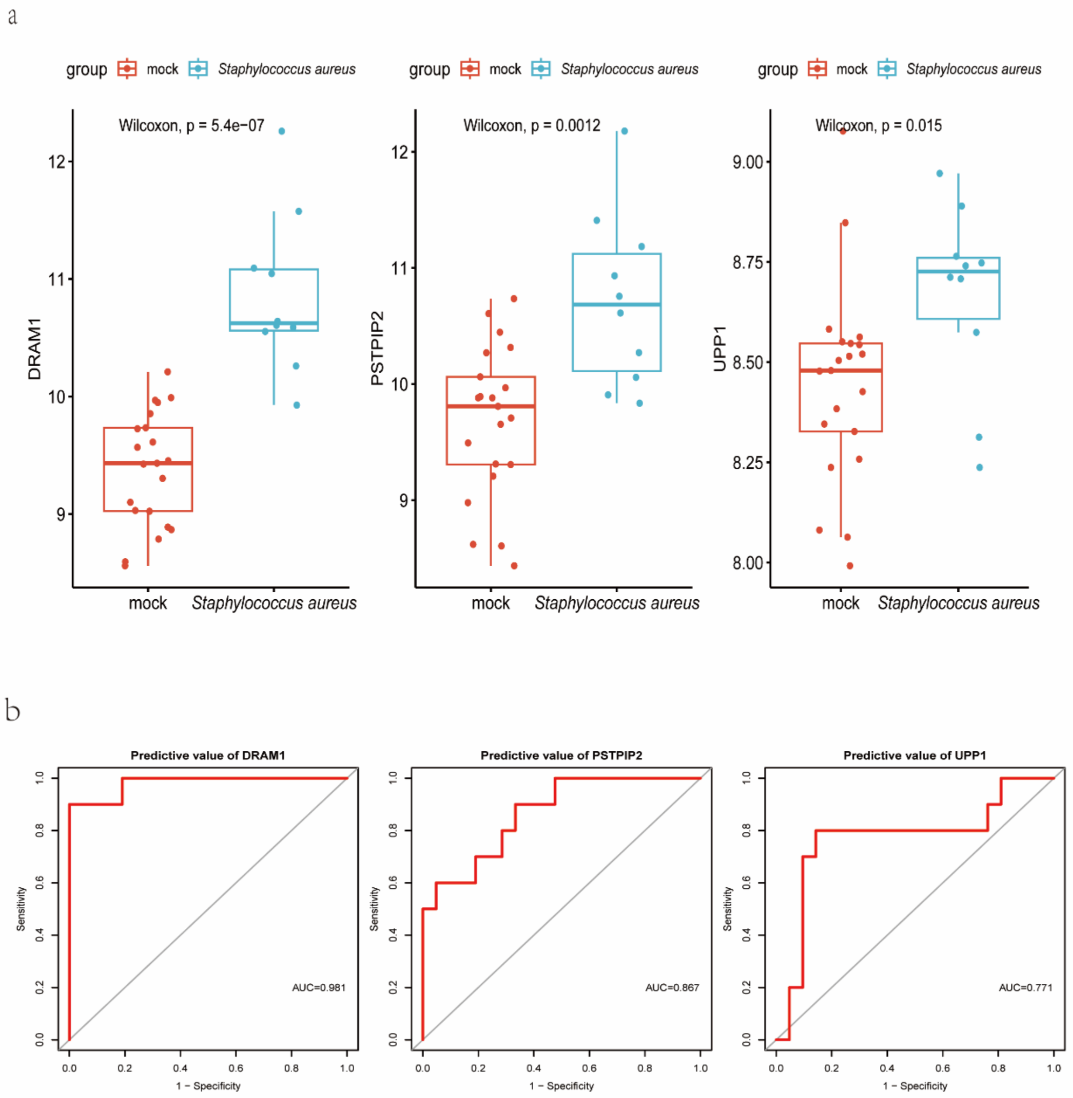
a
group mock Staphylococcus aureus
Wilcoxon, p = 5.4e−07
DRAM1
Wilcoxon, p = 0.0012
PSTPIP2
Wilcoxon, p = 0.015
UPP1
mock Staphylococcus aureus
b
Predictive value of DRAM1
AUC=0.981
Predictive value of PSTPIP2
AUC=0.867
Predictive value of UPP1
AUC=0.771
Sensitivity
1 − Specificity
